# A Variant of the *SLC10A2* Gene Encoding the Apical Sodium-Dependent Bile Acid Transporter Is a Risk Factor for Gallstone Disease

**DOI:** 10.1371/journal.pone.0007321

**Published:** 2009-10-13

**Authors:** Olga Renner, Simone Harsch, Elke Schaeffeler, Stefan Winter, Matthias Schwab, Marcin Krawczyk, Jonas Rosendahl, Henning Wittenburg, Frank Lammert, Eduard F. Stange

**Affiliations:** 1 Dr. Margarete Fischer-Bosch Institute of Clinical Pharmacology and University of Tuebingen, Stuttgart, Germany; 2 Department of Medicine II, Saarland University Hospital, Homburg, Germany; 3 Department of Gastroenterology and Hepatology, University of Leipzig, Leipzig, Germany; 4 Department of Internal Medicine I, Robert Bosch Hospital, Stuttgart, Germany; 5 Department Clinical Pharmacology, University Hospital Tuebingen, Tuebingen, Germany; Stanford University, United States of America

## Abstract

**Background:**

Cholelithiasis is a multifactorial process and several mechanisms of gallstone formation have been postulated. As one of these mechanisms, a decreased expression of the ileal apical sodium-dependent bile acid transporter gene *SLC10A2* in gallstone carriers was described previously. In this study the *SLC10A2* gene was investigated to identify novel genetic variants and their association with gallstone formation.

**Methodology/Principal Findings:**

Study subjects were selected with the presence or absence of gallstones confirmed by ultrasound and medical history. Genomic DNA was obtained from blood leukocytes. Sequence analysis was performed of all six exonic and flanking regions as well as of 2,400 base pairs of the *SLC10A2* promoter in a cohort of gallstone carriers and control subjects from Stuttgart, Germany. Genotype frequencies of newly identified genetic variants (n = 6) and known single nucleotide polymorphisms (n = 24) were established using MALDI-TOF mass spectrometry. Six new genetic variants were found within the *SLC10A2* gene. Although none of the variants was linked to gallstone disease in the Stuttgart cohort overall, the minor allele of SNP *rs9514089* was more prevalent in male non-obese gallstone carriers (*p* = 0.06680, OR = 11.00). In a separate population from Aachen, Germany, the occurrence of *rs9514089* was two-fold higher in gallstone patients (22%) than in corresponding controls (11%) (*p* = 0.00995, OR = 2.19). In the pooled Aachen/Stuttgart cohort *rs9514089* was highly significantly linked to cholelithiasis (*p* = 0.00767, OR = 2.04). A more frequent occurrence was observed for male gallstone carriers (22%) compared to controls (9%) (*p* = 0.01017, OR = 2.99), for the total normal weight group (*p* = 0.00754, OR = 2.90), and for male non-obese gallstone patients (*p* = 0.01410, OR = 6.85). Moreover, for the minor allele of *rs9514089* an association with low plasma cholesterol levels was found especially in gallstone carriers (*p* = 0.05).

**Conclusions/Significance:**

We have identified *SLC10A2* as a novel susceptibility gene for cholelithiasis in humans. Comprehensive statistical analysis provides strong evidence that *rs9514089* is a genetic determinant especially in male non-obese gallstone carriers. The minor allele of *rs9514089* is related to differences in plasma cholesterol levels among the subjects.

## Introduction

Cholelithiasis is the most common gastrointestinal disease with a prevalence of 15% in European adults [1]–[4]. Family and twin studies in humans as well as inbred mice have clearly demonstrated that a complex genetic basis determines the individual risk to develop cholesterol gallstones in addition to factors such as obesity and pregnancy [5]–[7].

Several chromosomal regions exhibit linkage to gallstone disease. Complementary to the murine model [8], [9], the detailed analysis of human gallstone susceptibility genes in Mexican families provided suggestive evidence of linkage on chromosome 1p [6]. Rosmorduc et al. [10] showed that rare loss of function mutations in the *ABCB4* gene encoding the hepatocanalicular transporter required for biliary phospatidylcholine secretion may lead to gallstone formation in certain patients. These findings in humans are consistent with the spontaneous occurrence of gallstones in *Abcb4* knockout mice [11]. An increased gallstone risk in humans was demonstrated for the *ABCG8* 19H allele by a genomewide association scan with 500,000 SNPs in Caucasian individuals [12]. These findings were also confirmed in the combined linkage and association study by Grünhage and co-workers [13]. Additionally, the results are consistent with supporting evidence from animal model that the *ABCG5/G8* transporter is a lithogenic risk factor [9]. Another recent association study observed an Arg^64^-variant of the β_3_-adrenergic receptor (*ADRB3*) more frequently in gallstone patients [14]. Moreover, variations of the gene encoding the nuclear bile salt receptor FXR in an inbred mouse model and in three ethnically distinct populations were described as risk factors for the development of cholelithiasis [15].

In the current study we follow up on our previous work that investigated a role of the apical sodium-dependent bile acid transporter (ASBT, gene name *SLC10A2*) in gallstone formation. We have shown that in non-obese gallstone patients, ASBT expression is diminished, and that it is associated with low cytosolic ileal lipid binding protein (ILBP) and basolateral organic solute bile acid exporter (OSTα-OSTβ) expression [16], [17]. In line with differences in cholesterol metabolism in lean and obese subjects [18], the reduced expression of the transporter was weight-specific and observed in normal weight individuals only [16]. Furthermore, subclinical inflammation was excluded as the molecular pathomechanism of reduced ASBT expression in gallstone patients [19]. The role of this transporter has been suggested to be the major determinant of bile acid pool size [20]. The size of the circulating pool of bile acids is an essential factor influencing cholesterol homeostasis [21]. Moreover, an impaired intestinal bile acid absorption, resulting in fecal bile acid loss and a decreased bile acid pool size, was identified to represent the primary defect in some patients with cholesterol gallstones [22]. Accordingly, a reduction of the bile acid pool size and the cholesterol supersaturation of gallbladder bile were identified as pivotal pathophysiological defects that promote cholesterol gallstone formation [23]–[27].

Based on these findings we hypothesized that mutations of the *SLC10A2* gene might result in gallstone formation by impairing the bile acid pool size. The first human mutation rendering *SLC10A2* dysfunctional was identified in a patient with Crohn's disease [28]. Moreover, it was shown that inherited mutations within the human *SLC10A2* gene are responsible for the abolished bile acid transport in a family with congenital primary bile acid malabsorption (PBAM) [29]. In addition, the *SLC10A2* region was suggested to be relevant in bilirubin metabolism in a genome wide screening study [30]. Also, the frame-shift mutation 646insG in *SLC10A2*, which was associated with the abolished bile acid transport activity, was detected in a patient with familial hypertriglyceridemia (FHTG) [31]. In addition to genetic investigations, impaired intestinal bile acid absorption was observed in patients with hypertriglyceridemia as a result of diminished expression of apical bile acid transporter protein ASBT [32]. Recently, we demonstrated a reduced expression of ASBT that was linked to a haplotype block of *SLC10A2* containing a total of ten variants [33].

The aim of the present study was to systematically analyze the *SLC10A2* gene for genetic variants and to investigate whether the variants are associated with the development of gallstones. We established the genotype frequencies of a total of 30 sequence variations within the *SLC10A2* gene; six of these variants were newly identified. Most importantly, an association of the SNP *rs9514089* with gallstone prevalence was observed in two distinct human Caucasian populations suggesting that a genetic variant of *SLC10A2* confers an increased risk of gallstone formation.

## Results

### Sequence Analysis of *SLC10A2*

To identify novel genetic variants associated with cholelithiasis, the *SLC10A2* gene was screened by sequencing, using a total of 93 samples from the Stuttgart population. The mutational analysis of 60 gallstone carriers and 33 control individuals resulted in the identification of six novel distinct variants in the untranslated region (5′-UTR) and in the coding regions of *SLC10A2*. These genetic variants (two in the 5′ UTR region, two in exon 1 and two in exon 5) were found in female and in male gallstone carriers as well as in one male control. All individuals carried the newly identified variants in the heterozygous state. Detailed information of the variants is listed in Table 1.

**Table 1:** Main characteristics of all 30 SNPs examined in the Stuttgart population.

Variant	dbSNP	Region	cDNA a/Genotype	Protein b	MAF c	HWE
	rs/ss number					(*p-*value) d
**1**	rs3759501	upstream of 5′-UTR	T>**A**		0.343	1.0
**2**	rs3759502	upstream of 5′-UTR	C>**T**		0.337	1.0
**3**	rs3759503	upstream of 5′-UTR	C>**T**		0.346	1.0
**4**	rs3759504	upstream of 5′-UTR	T>**C**		0.323	0.6092
**5**	rs7990819 e	upstream of 5′-UTR	T>**C**		0.044	1.0
**6**	rs66994812 e	upstream of 5′-UTR	C>**T**		0.037	1.0
**7**	rs66924010 e	upstream of 5′-UTR	T>**C**		0.043	1.0
**8**	rs71653645 f	5′-UTR	c.-548C>**T**		0.0040	1.0
**9**	rs71653646 f	5′-UTR	c.-332C>**T**		0.0040	1.0
**10**	rs16961281	5′-UTR	c.-225C>**T**		0.067	0.8779
**11**	rs41281682 e	5′-UTR	c.-17C>**G**		0.043	1.0
**12**	rs41281680 e	exon 1	c.129C>**T**	p.A43	0.043	1.0
**13**	rs71640246 f	exon 1	c.156C>**T**	p.N52	0.0080	1.0
**14**	rs71640247 f	exon 1	c.197G>**A**	p.W66X	0.0040	1.0
**15**	rs55971546	exon 1	c.292G>**A**	p.V98I	0.043	1.0
**16**	ss99307920 e, g	exon 1/intron 1 boundary	c.377+12T>**C**		0.043	1.0
**17**	rs1329516	exon 1/intron 1 boundary	c.377+109G>**T**		0.0	1.0
**18**	rs9514089	intron 1/exon 2 boundary	c.378-105A>**G**		0.366	1.0
**19**	ss99307921 e, g	intron 1/exon 2 boundary	c.378-97A>**G**		0.037	1.0
**20**	rs157381	exon 2	c.426G>**A**	p.P142	0.0	1.0
**21**	rs60380298 e	exon 2	c.475G>**A**	p.V159I	0.043	1.0
**22**	rs67736127 e	intron 2/exon 3 boundary	c.497-74C>**A**		0.043	1.0
**23**	rs66842575 e	intron 2/exon 3 boundary	c.497-40A>**T**		0.043	1.0
**24**	rs41281678	exon 3	c.505C>**T**	p.L169	0.031	0.2148
**25**	rs188096	exon 3	c.511G>**T**	p.A171S	0.142	0.9408
**26**	-	exon 4	c.728T>**C**	p.L243P	0.0	1.0
**27**	rs72547505	exon 5	c.785C>**T**	p.T262M	0.0	1.0
**28**	rs56398830	exon 5	c.868C>**T**	p.P290S	0.02	1.0
**29**	rs71640248 f	exon 5	c.886T>**C**	p.F296L	0.0040	1.0
**30******	rs61966074 f	exon 5	c.910T>**C**	p.F304L	0.0080	1.0

SNPs are numbered 1–30 reflecting their sequential order on the physical map of *SLC10A2* sequence.

5′-UTR = untranslated region.

**^a^**Numbering according to transcript NM_00452 including the transition initiation codon.

**^b^**Numbering according to NP_00443.1 starting at translation initiation codon.

**^c^**Minor allele frequencies (MAF) are indicated in bold.

**^d^**Hardy-Weinberg equilibrium.

**^e^**Polymorphism was previously described to be associated with reduced *SLC10A2*-expression [32].

**^f^**Newly identified genetic variant.

**^g^**rs-number is available with the next dbSNP Build, B131 (planned for November 2009).

### Genetic Variations of *SLC10A2* in the Stuttgart Cohort

To investigate whether any of the variants were associated with gallstones, we compared the genotype frequencies of all single nucleotide polymorphisms (SNPs) in gallstone carriers and control subjects in the Stuttgart cohort. In addition to the newly identified genetic variants, based on frequency, 24 previously reported SNPs of *SLC10A2* were selected from the NCBI data bases (http://www.ncbi.nlm.nih.gov/sites/entrez) to explore their association with cholelithiasis. The main characteristics of all 30 SNPs examined in the Stuttgart population are listed in Table 1. The study population was subdivided into weight-, gender- and disease-specific subgroups and genotype frequencies, odds ratios (ORs) and confidence intervals (CI) for each subgroup were calculated to obtain genotype-associated disease risk estimations. Moreover, the dominant and recessive models of inheritance were tested by analyzing wild type homozygous individuals (MM) versus all carriers of the minor variant (mm) or by comparison of wild type homozygous and heterozygous (Mm) individuals to the subjects with the minor allele ((MM+Mm)< >mm). Generally, in a total of 127 genotyped individuals the frequencies of most common SNPs showed neither significant gender-specific nor weight-specific differences. The overall minor allele frequencies (MAF) of the *SLC10A2* SNPs ranged from 0 to 0.366. The majority of these SNPs displayed MAF lower than 10% in the population analyzed.

The polymorphisms of the promoter and untranslated regions (UTR) (*rs3759501*, *rs3759502*, *rs3759503*, *rs3759504*, *rs7990819*, *rs16961281*, *rs41281682*) were detected in gallstone carriers and controls, and their distributions did not exhibit any significant differences between both groups. The novel genetic variants *rs71653645*/c.-548C>T and *rs71653646*/c.-332C>T, that were identified in two separate gallstone carriers, were not found in any additional subjects. The synonymous variant *rs71640246*/p.N52 in exon 1 was identified in two male gallstone carriers only. Moreover, the female gallstone carrier with the nucleotide change G-to-A leading to the premature stop codon at position 66 (*rs71640247*/p.W66X) represented a single case of individual sequence alteration. Notably, in our cohort no variant alleles were found for the following four sequence variants that were described in the database: intron 1: *rs1329516*, exon 2: *rs157381*/Pro142, exon 5: L243P, *rs72547505*/T262M. In addition, there were no statistical differences between gallstone carriers and controls for the various coding region variants: *rs41281680*/p.A43, *rs55971546*/A98I *rs60380298*/V159I, r*s41281678*/L169, *rs188096*/A171S. The mutation P290S *(rs56398830)* rendering *SLC10A2* dysfunctional, which was identified initially in a patient diagnosed with Crohn's disease [28], was present at higher frequency in the control population (5.2%) compared to gallstone carriers (2.6%, *p* = 0.3534, OR = 0.6393). Moreover, this SNP occurred more frequently in overweight individuals (6.9%) compared to non-obese subjects (1.7%) (*p* = 0.0638, OR = 3.082). Furthermore, the newly identified sequence alteration p.F296L was only present in the control group with a frequency of 1.4%. On the other hand, the distribution of the variant p.F304L was similar between the case (1.8%) and control (1.4%) groups.

Interestingly, for one of the sequence variants, *rs9514089*, we observed a trend towards an association with cholelithiasis in this Stuttgart population. However, this association was observed only in male gallstone carriers with normal weight (20%) compared to male normal weight controls (0%) (data shown as supplemental Table S1).

### *SLC10A2* Polymorphism *rs9514089* in the Aachen Cohort

To confirm the association of the *rs9514089* polymorphism to gallstones, a larger additional population from Aachen, comprising matched pairs of gallstone carriers and controls, was analyzed. Interestingly, the Aachen cohort expressed a clearly higher SNP frequency of the *rs9514089* variant in gallstone carriers of the total population as well as in every distinct subgroup compared with control subjects. In the total population, this association was significant (*p* = 0.00995, OR = 2.19). In this population, the G allele was more frequent in gallstone carriers (22%) compared to controls (11%). Moreover, the significant association of the minor allele with gallstone prevalence was also observed in male gallstone carriers (*p* = 0.03319, OR = 2.79) as well as in normal weight gallstone patients (*p* = 0.03517, OR = 2.59) (data shown as supplemental Table S2). The *p-*values and risk estimations for the Aachen cohort and the pooled population are listed for the recessive model.

### *SLC10A2* Polymorphism *rs9514089* in the Pooled Population

To increase the power of our analysis, the populations of Stuttgart and Aachen were pooled. For this analysis, the distribution of the SNP *rs9514089* was determined in a total of 255 controls and 240 gallstone carriers (Table 2). In the entire pooled cohort, we observed an association of *rs9514089* with gallstone carrier status (*p* = 0.00767, OR = 2.04). The increased frequency of the homozygous GG genotype in the combined analysis of all samples from both independent German cohorts was observed for all gallstone patients (GG frequency 20%) in comparison to the control group (GG frequency 11%). In accordance with our results in the individual single cohorts, the pooled population also demonstrated a more frequent occurrence (*p* = 0.01017, OR = 2.99) of the genetic variant in male gallstone carriers (22%) compared to male controls (9%). Furthermore, the analysis of the different gallstone carrier subgroups revealed a significant association of the minor variant with gallstone susceptibility in the normal weight group (*p* = 0.00754, OR = 2.90). In contrast to female normal weight gallstone carriers, an increased SNP frequency was observed in the male non-obese gallstone patients in comparison to the male non-obese control group (*p* = 0.01410, OR = 6.85).

**Table 2:** Prevalence of gallstones and *rs9514089* polymorphism in the pooled Stuttgart and Aachen cohorts.

Subgroup	Controls n (%)	Gallstone carriers n (%)	AA a< >gg c	(AA a+Ag b)< >gg c
Genotype	A/A	A/G	G/G	A/A	A/G	G/G	*p-*value	OR (95% CI)	*p-*value	OR (95% CI)
**Total**	93 (37)	129 (52)	27 (11)	91 (38)	98 (42)	47 (20)	0.05303	1.78 (0.99–3.23)	**0.00767**	**2.04 (1.19–3.55)**
**Males**	47 (40)	59 (51)	10 (9)	40 (42)	34 (36)	21 (22)	0.05864	2.45 (0.97–6.55)	**0.01017**	**2.99 (1.26–7.56)**
**Females**	50 (37)	70 (51)	17 (12)	51 (36)	64 (45)	26 (19)	0.36153	1.50 (0.69–3.33)	0.18639	1.59 (0.78–3.31)
**Normal weight ^e^**	49 (38)	69 (53)	11 (9)	40 (39)	41 (40)	22 (21)	**0.04184**	**2.43 (0.99–6.27)**	**0.00754**	**2.90 (1.27–7.01)**
**Males**	22 (43)	27 (53)	2 (4)	17 (47)	11 (31)	8 (22)	0.07375	5.01 (0.85–54.42)	**0.01410**	**6.85 (1.25–70.70)**
**Females**	27 (35)	42 (54)	9 (11)	23 (34)	30 (45)	14 (21)	0.31514	1.81 (0.60–5.71)	0.17103	2.02 (0.75–5.71)
**Overweight ^f^**	44 (37)	60 (50)	16 (13)	51 (38)	57 (43)	25 (19)	0.45777	1.35 (0.60–3.07)	0.30547	1.50 (0.72–3.20)
**Males**	25 (39)	32 (49)	8 (12)	23 (39)	23 (39)	13 (22)	0.30883	1.75 (0.55–5.85)	0.16034	2.00 (0.70–6.09)
**Females**	19 (35)	28 (51)	8 (14)	28 (38)	34 (46)	12 (16)	1.00000	1.02 (0.31–3.46)	1.00000	1.14 (0.39–3.48)

*p*<0.05 was regarded as statistically significant, odds ratio (OR) and 95% confidence interval (CI).

a AA = major allele.

b Ag = heterozygote allele.

c gg = minor allele, the subjects were divided into subgroups with body mass index (BMI)≤25 kg/m^2^ = **^e^** normal weight and BMI>25 kg/m^2^ = **^f^** overweight.

### *rs9514089* GG Genotype Frequencies in *SLC10A2* and Plasma Lipid Levels

The distributions *of rs9514089* GG genotypes in both Stuttgart and Aachen populations as well as in the pooled group were analyzed with respect to age, BMI, plasma cholesterol and triglyceride levels (Table S3). For the pooled group, a correction term for the study centre was incorporated in the models. The various genotypes were not associated with age, BMI or triglycerides. However, patients carrying the G allele of *rs9514089* displayed lower total cholesterol levels compared to patients with the A allele. In the association analysis, a significant association of *rs9514089* and total cholesterol levels was observed in the Aachen cohort (*p* = 0.04, data not shown). The Stuttgart cohort demonstrated a non-significant trend towards lower cholesterol plasma levels in gallstone patients with GG genotypes than in patients with the major allele. In the pooled population, we confirmed the association of the GG genotypes of *rs9514089* in gallstone carriers with low cholesterol levels. After correction by study centre, this association was only observed to display a borderline effect (*p* = 0.05).

## Discussion

In this candidate gene study the *SCL10A2* gene was systematically sequenced, and a first cohort from Stuttgart was genotyped for the association of a total of 30 SNPs with cholelithiasis. Among the analyzed variants, the SNP *rs9514089* was significantly more prevalent in male non-obese gallstone carriers. Subsequently, this association was verified in an additional larger replication cohort. In addition this SNP was associated with lower plasma cholesterol values.

This is the first positive genetic association study of a factor involved in bile acid transport. Previous association studies have reported a linkage of gallstone formation and point mutations in the *ABCB4* gene encoding the phospholipid transporter MDR3 [10], [34]. This is consistent with the spontaneous occurrence of cholelithiasis in *Abcb4* knockout mice [11]. A genomewide association scan has linked the coding variant D19H of the *ABCG8*, the hepatocanalicular cholesterol transporter, to this disease with an OR of 2.2 [12]. This association was confirmed by Grünhage et al. in a nonparametric linkage analysis of affected sib pairs with the 19H allele compared to gallstone-free controls (OR = 3.0) [13]. The study of Klass et al. identified a gallstone-associated polymorphism in the hepatic β3-adrenergic receptor (ADRB3) suggested to affect gallbladder motility [14]. Kovacs et al. described a linkage of variations of the FXR gene and gallstone susceptibility in humans [15]. Notably, the linkage to cholelithiasis was gender-specific (males: OR = 3.09; females: OR = 0.85). No association of this haplotype with gallstone prevalence could be found in a German cohort (OR = 0.83) and, in a Chilean female population a paradoxical trend towards a protective effect of *NR1H4_1* was observed (*p* = 0.08, OR = 0.69).

So far no gallstone-associated SNP has been described for bile acid transporters in the intestine. For the *SLC10A2* gene only a few dysfunctional mutations were identified in single patients diagnosed with Crohn's disease, hypertriglyceridemia and/or in patients with different forms of bile acid malabsorption [28], [31], [35], [36]. However, these loss-of-function-mutations could not be associated with specific diseases in general. In the current study, we identified the polymorphism *rs9514089* of *SLC10A2* as the first intestinal bile acid related candidate for the development of gallstones. In the pooled cohort from Stuttgart and Aachen we found a significant association of the polymorphism *rs9514089* with gallstone disease (*p* = 0.00767, OR = 2.04). In addition, we analyzed the effect of the SNP *rs9514089* in different gender- and weight-specific groups. Herein, this sequence variant was significantly associated with cholelithiasis in the total male and in the total normal weight subgroups. However, the most significant linkage was found for male normal weight gallstone carriers (*p* = 0.01410, OR = 6.85), indicating that the occurrence of the *rs9514089* polymorphism is gender- and weight-specific.

Although our study demonstrates, that the genetic gallstone disease risk is attributable to the GG variant in the *SLC10A2* gene, it remains unclear how this genetic variant affects the function of the ASBT transporter and how these functional differences lead to gallstone disease. Several scenarios may possibly explain the different prevalence of this polymorphism in gallstone carriers and controls: First, in carriers of the G allele, the SNP *rs9514089* could be the relevant polymorphism, causing a decreased transcription of the gene that could lead to a diminished epithelial bile acid uptake. Second, it may be hypothesized that the genomic structure of the *SLC10A2* gene with the G allele is the major determinant of the diminished ASBT expression though post-transcriptional mechanisms could influence protein expression. Third, the GG variant could alter the binding of several transcription factors promoting gallstone formation. As mentioned above, we have previously shown that expression of this transporter is diminished in the ileum of normal weight gallstone patients [16]. Unfortunately, our cohort of patients from which we were able to obtain ileal biopsies is too small for genetic association studies.

To assess whether the association results for the GG variant of the *SLC10A2* gene were affected by confounding risk factors for gallstones, we performed a multivariate analysis that included the *rs9514089* variant and lithogenic risk factors such as age, gender, BMI and plasma cholesterol levels. The only significant association relates to serum cholesterol in the Aachen population, where lower cholesterol levels, were found in gallstone carriers of the GG genotype of the *rs9514089* variant compared with gallstone carriers with the major allele. Although this trend was confirmed in the Stuttgart cohort, in the pooled population the association of the GG genotype in gallstone carriers with low cholesterol levels was only borderline (*p* = 0.05). Such an association with a lipid transporter is in line with prior studies where the D19H and T400K variants of *ABCG8* and the *ABCG5* Q604E polymorphism were associated with altered plasma cholesterol levels [37]–[39]. On the other hand, Acalovschi et al. found no link between *ABCB4* and *ABCB11* polymorphisms, lithogenic dyslipidemia, and gallstone risk [40]. Furthermore, in previous studies low total cholesterol levels have been linked to the development of gallstones [41] but in our cohorts this was not the case. Therefore, and also because of the lack of significance in the total cohort, there is little evidence for a confounder effect of serum cholesterol in the present investigation. There was also no relevant association between the *rs9514089* variant and triglyceride levels, although patients with hypertriglyceridemia have been demonstrated to exhibit diminished expression of apical sodium-dependent bile acid transporter protein [32]. To assess the association of *rs9514089* polymorphism to plasma lipids further, future investigations in additional populations are required.

In conclusion, we identified *SLC10A2* as a novel intestinal common susceptibility factor for gallstones in humans. The results of this study may prompt investigations which will address more comprehensive genotype-phenotype association. In the future, additional genetic risk factors are likely to be identified, which we envisage to provide novel means for the risk assessment and possibly prevention of gallstones.

## Materials and Methods

### Ethics statement

The local ethics comittees (for the study population from Aachen: ethics committees from the Departement of Medicine University of Aachen and Departement of Medicine University of Leipzig as well as for the study population from Stuttgart: ethics committee of the University Hospital of Tuebingen and University Tuebingen) approved these studies and all subjects gave written informed consent prior to participation.

### NCBI data statement

All new data have been deposited in GenBank (NCBI data bank http://www.ncbi.nlm.nih.gov/BankIt), rs-numbers are already available.

### Study populations and clinical characteristics

The study population from Stuttgart (Germany), comprises 127 individuals, of which 71 are healthy individuals and 56 are gallstone carriers. The recruitment criteria for the subjects included in this study were described previously [16], [17], [33]. The clinical characteristics of the study participants, including age, BMI, triglyceride and cholesterol levels did not differ significantly between the gallstone carriers and controls and are summarized in Table 3.

**Table 3:** Clinical characteristics of study participants from Stuttgart and Aachen cohorts.

Variables	Stuttgart controls (n = 71)	Stuttgart gallstone carriers (n = 56)	Aachen controls (n = 184)	Aachen gallstone carriers (n = 184)
**Gender (f/m)**	*(33/38)*	*(40/16)*	*(103/81)*	*(103/81)*
**Age (years)**	*60±12*	*63±12*	*63±13*	*63±13*
**BMI (kg/m^2^)**	*26±4*	*26±4*	*25.4±4.1*	*26.1±4.3*
**Triglyceride (mg/dl) (1–200)** a	*128±82*	*132±92*	*146±105*	*143±79*
**Cholesterol (mg/dl) (140–240)** a	*200±34*	*198±37*	*202±55*	*200±53*

Values are given as means±SD; BMI = body mass index;

a = normal range.

The characteristics of the Aachen population were, in part, published previously [42].

In the study population from Aachen (Germany), including 368 subjects with 184 matched pairs for age and BMI, the presence of gallstones was diagnosed by ultrasound or history of prior cholecystectomy [42]. Characteristics of the cohort are summarized in Table 3.

The Caucasian origin of both cohorts was determined by name and personal interview.

### Mutational analysis of *SLC10A2* gene

Based on the genomic sequence (Gene Bank Accession number: NC_000013.9), oligonucleotide primer sets were designed spanning all six exons of *SLC10A2*, the intron/exon junctions (50–100 nucleotides) as well as a 2,400 base pairs (bp) promoter fragment. Primers were created using Primer3 program (http://frodo.wi.mit.edu/), the quality control was performed with Oligonucleotide Properties Calculator (http://www.basic.northwestern.edu/biotools/oligocalc.html), and the uniqueness in the genome was confirmed applying BLAST software (http://blast.ncbi.nlm.nih.gov/Blast.cgi). For PCR reactions, the HotStarTaq (QIAGEN) and FastStart High Fidelity (Roche) DNA polymerase systems were used according to manufacturer's instructions. All PCR amplification reactions were performed on DYAD™ PCR thermal cyclers (MJ Research). PCR products were split into two different tubes to perform the forward and reverse amplifications. The sequencing PCR was performed using specific nested primers, according to standard procedures (Big Dye® Terminator Mix v 1.1, Applied Biosystems). DNA sequencing of both strands was performed by GENterprise Genomics (Mainz, Germany), followed by analysis on an ABI Prism 310 automated Genetic Analyzer (Applied Biosystems). Novel genetic variations were confirmed by repeated sequencing of PCR products in both directions. The nomenclature of the new variants follows the recommendations for the description of DNA changes by Dunnen and Antonarakis [43].

### Genotyping

Analysis of SNPs was performed using matrix-assisted laser desorption/ionization time-of-flight mass spectrometry (MALDI-TOF MS) of allele specific primer extension products. Genotype frequencies of the variants were established using two separate assays. All reactions, including PCR amplification, shrimp alkaline phosphatase treatment, and base extension were performed in 384-well plates (ABgene) with a Puredisk pipette robot (Cybio). PCR amplification and primer extension reaction were carried out in a DYAD™ PCR thermal cycler (MJ Research). For quality control we performed no-template controls in all plates and repeated analysis of 10% of randomly selected samples.

In the study population from Aachen, genotyping of *rs9514089* was performed using a TaqMan allelic discrimination assay (Custom TaqMan SNP Genotyping Assay; Applied Biosystems, Inc.). Oligonucleotide sequences of probes and PCR primers are available upon request. The TaqMan genotyping reaction was amplified (95°C for 10 min, and 92°C for 15 sec, and 60°C for 1 min, for 40 cycles) and fluorescence was detected on the ABI PRISM 7700 sequence detection system (Applied Biosystems, Inc.). To assess reproducibility, a random ∼5% selection of the sample was re-genotyped and all of these genotypes matched with genotypes initially designated.

### Multiplex reaction

Five ng of genomic DNA were amplified by PCR, containing locus-specific primers and HotStarTaq DNA polymerase (QIAGEN). PCR conditions were 95°C for 15 minutes for hot start, followed by 44 cycles of denaturation at 95°C for 30 seconds, annealing at 56°C for 1 minute, extension at 72°C for 1 minute and finally incubation at 72°C for 10 minutes. Subsequently, PCR products were treated with shrimp alkaline phosphatase (iPLEX™ Gold Kit, SEQUENOM) at 37°C for 20 minutes to remove excess of deoxynucleotide triphosphates, followed by 10 minutes at 85°C to inactivate the enzyme.

### Extension reactions

Purified PCR products were used as templates for extension reactions with extension primers and iPLEX enzyme (iPLEX™ Gold Kit, SEQUENOM) following the instructions of the supplier. The iPLEX reaction was performed using two-step 200 short cycles program (94°C, 30 seconds; 40 x (94°C, 5 seconds; 5 x (52°C, 5 seconds; 80°C, 5 seconds)); 72°C, 3 minutes). The final base extension products were treated with a cationic exchange resin to remove salts. Reaction solutions were dispensed onto the 384-well format SpectroCHIP® Array chips (SEQUENOM) and mass spectrometric analyses were performed on a Compact Mass Spectrometer. Data were analyzed using the Typer software (both SEQUENOM).

Primer sequences for sequence analyses, multiplex reactions and extension reactions are available upon request.

### Computational methods and statistical analysis

Clinical characteristics of study participants are expressed as means ± (SD). Observed and expected allele and genotype frequencies within the study population were compared by means of Hardy–Weinberg equilibrium calculations [44]. Data were considered in Hardy–Weinberg equilibrium when *p-*values were >0.05. Statistical analysis of differences between gallstone carriers and controls was done using Fisher's exact and Chi-square tests. Risk estimations for the development of gallstones were calculated as odds ratios (ORs) together with a 95% confidence interval (CI). Associations of clinical parameters and *rs9514089* polymorphism, corrected for study centre (Aachen or Stuttgart), were analyzed with library SNPassoc (version 1.5–8) [45] of the statistical software R-2.8.1 (http://www.r-project.org). Library SNPassoc was also applied in order to assess the effect of the *rs9514089* polymorphism on the development of gallstones in multivariate models. Here, we accounted for the adjustment factors age, gender, body mass index (BMI) and plasma cholesterol levels. Moreover, for the pooled population, a correction term for the study centres was included. All statistical tests were two-tailed and a *p-*value of <0.05 was considered as statistically significant.

## Supporting Information

Table S1 Prevalence of gallstones and rs9514089 polymorphism in the Stuttgart cohort. p<0.05 was regarded as statistically significant, odds ratio (OR) and 95% confidence interval (CI), a AA = major allele, b Ag = heterozygous allele, c gg = minor allele, the subjects were divided into subgroups with body mass index, (BMI)≤25 kg/m^2^ = e normal weight and BMI>25 kg/m^2^ = f overweight.
(0.04 MB DOC)

Table S2 Prevalence of gallstones and rs9514089 polymorphism in the Aachen cohort. p<0.05 was regarded as statistically significant, odds ratio (OR) and 95% confidence interval (CI), a AA = major allele, b Ag = heterozygous allele, c gg = minor allele, the subjects were divided into subgroups with body mass index, (BMI)≤25 kg/m^2^ = e normal weight and BMI>25 kg/m^2^ = f overweight.
(0.04 MB DOC)

Table S3 Clinical parameters in pooled Stuttgart and Aachen controls and gallstone carriers with distinct rs9514089 genotypes. Values are given as means±SEM; BMI = body mass index; a = normal range; b = in the recessive model, corrected by study centre.
(0.04 MB DOC)
