# Supplementary material for: A Variant of the SLC10A2 Gene Encoding the Apical Sodium-Dependent Bile Acid Transporter Is a Risk Factor for Gallstone Disease
Source: PLoS One. 2009 Oct 13;4(10):e7321. doi: 10.1371/journal.pone.0007321 (PMC2757911; doi:10.1371/journal.pone.0007321)
Supplement: Table S1 — Prevalence of gallstones and rs9514089 polymorphism in the Stuttgart cohort. p<0.05 was regarded as statistically significant, odds ratio (OR) and 95% confidence interval (CI), a AA = major allele, b Ag = heterozygous allele, c gg = minor allele, the subjects were divided into subgroups with body mass index, (BMI)≤25 kg/m2 = e normal weight and BMI>25 kg/m2 = f overweight. (0.04 MB DOC) [file pone.0007321.s001.doc]

**Supplemental: Table S1.** Prevalence of gallstones and *rs9514089* polymorphism in the Stuttgart cohort

| **Subgroup** |  | **Controls** | **n (%)** | **Gallstone** | **carriers** | **n (%)** |  | **AA ^a^< >gg ^c^** |  | **(AA ^a^+Ag ^b^)< >gg ^c^** |
| --- | --- | --- | --- | --- | --- | --- | --- | --- | --- | --- |
| **Genotype** | **A/A** | **A/G** | **G/G** | **A/A** | **A/G** | **G/G** | *p-*value | OR (95% CI) | *p-*value | OR (95% CI) |
| **Total** | 31 (44) | 32 (46) | 7 (10) | 21 (38) | 27 (48) | 8 (14) | 0.37255 | 1.69 (0.53-5.36) | 0.46042 | 1.50 (0.51-4.43) |
| **Males** | 18 (49) | 17 (46) | 2 (5) | 7 (44) | 7 (44) | 2 (12) | 0.37721 | 2.57 (0.30-22.00) | 0.36938 | 2.50 (0.32-19.54) |
| **Females** | 13 (39) | 15 (46) | 5 (15) | 14 (35) | 20 (50) | 6 (15) | 0.88012 | 1.11 (0.27-4.55) | 0.98563 | 0.99 (0.27-3.58) |
| **Normal weight ^e^** | 15 (39) | 21 (55) | 2 (5) | 7 (32) | 11 (50) | 4 (18) | 0.12132 | 4.29 (0.63-29.24) | 0.10797 | 4.00 (0.67-23.95) |
| **Males** | 8 (50) | 8 (50) | - | 1 (20) | 3 (60) | 1 (20) | **0.03501** | **17.00 (0.45-648.79)** | **0.06680** | **11.00 (0.38-318.87)** |
| **Females** | 7 (32) | 13 (59) | 2 (9) | 6 (35) | 8 (47) | 3 (18) | 0.59873 | 1.75 (0.22-14.23) | 0.42804 | 2.14 (0.32-14.55) |
| **Overweight ^f^** | 16 (50) | 11 (34) | 5 (16) | 14 (41) | 16 (47) | 4 (12) | 0.90663 | 0.91 (0.20-4.09) | 0.64787 | 0.72 (0.18-2.96) |
| **Males** | 10 (48) | 9 (43) | 2 (9) | 6 (55) | 4 (36) | 1 (9) | 0.89080 | 0.83 (0.06-11.28) | 0.96817 | 0.95 (0.08-11.81) |
| **Females** | 6 (55) | 2 (18) | 3 (27) | 8 (35) | 12 (52) | 3 (13) | 0.76857 | 0.75 (0.11-5.11) | 0.30859 | 0.40 (0.07-2.42) |

*p*<0.05 was regarded as statistically significant, odds ratio (OR) and 95% confidence interval (CI), **^a^** AA = major allele,

**^b^** Ag = heterozygous allele, **^c^** gg = minor allele, the subjects were divided into subgroups with body mass index,

(BMI)≤25 kg/m² = **^e^** normal weight and BMI>25 kg/m² = **^f^** overweight.

(Renner et al.)
