# Supplementary material for: A Variant of the SLC10A2 Gene Encoding the Apical Sodium-Dependent Bile Acid Transporter Is a Risk Factor for Gallstone Disease
Source: PLoS One. 2009 Oct 13;4(10):e7321. doi: 10.1371/journal.pone.0007321 (PMC2757911; doi:10.1371/journal.pone.0007321)
Supplement: Table S2 — Prevalence of gallstones and rs9514089 polymorphism in the Aachen cohort. p<0.05 was regarded as statistically significant, odds ratio (OR) and 95% confidence interval (CI), a AA = major allele, b Ag = heterozygous allele, c gg = minor allele, the subjects were divided into subgroups with body mass index, (BMI)≤25 kg/m2 = e normal weight and BMI>25 kg/m2 = f overweight. (0.04 MB DOC) [file pone.0007321.s002.doc]

**Supplemental: Table S2. Prevalence of gallstones and *rs9514089* polymorphism in the Aachen cohort**

| **Subgroup** |  | **Controls** | **n (%)** | **Gallstone** | **carriers** | **n (%)** |  | **AA ^a^< >gg ^c^** |  | **(AA ^a^+Ag ^b^)< >gg ^c^** |
| --- | --- | --- | --- | --- | --- | --- | --- | --- | --- | --- |
| **Genotype** | **A/A** | **A/G** | **G/G** | **A/A** | **A/G** | **G/G** | *p-*value | OR (95% CI) | *p-*value | OR (95% CI) |
| **Total** | 62 (35) | 97 (54) | 20 (11) | 70 (39) | 71 (39) | 39 (22) | 0.11380 | 1.72 (0.87-3.47) | **0.00995** | **2.19 (1.19-4.17)** |
| **Males** | 29 (37) | 42 (53) | 8 (10) | 33 (42) | 27 (34) | 19 (24) | 0.16356 | 2.07 (0.73-6.33) | **0.03319** | **2.79 (1.07-7.92)** |
| **Females** | 33 (33) | 55 (55) | 12 (12) | 37 (37) | 44 (43) | 20 (20) | 0.39715 | 1.48 (0.58-3.87) | 0.17675 | 1.81 (0.78-4.33) |
| **Normal weight ^e^** | 34 (37) | 48 (53) | 9 (10) | 33 (41) | 30 (37) | 18 (22) | 0.17034 | 2.04 (0.74-5.96) | **0.03517** | **2.59 (1.02-7.01)** |
| **Males** | 14 (40) | 19 (54) | 2 (6) | 16 (52) | 8 (26) | 7 (22) | 0.26173 | 2.98 (0.46-34.05) | 0.07189 | 4.70 (0.80-50.29) |
| **Females** | 20 (36) | 29 (52) | 7 (12) | 17 (34) | 22 (44) | 11 (22) | 0.39112 | 1.83 (0.51-6.93) | 0.20814 | 1.96 (0.63-6.57) |
| **Overweight ^f^** | 28 (32) | 49 (56) | 11 (12) | 37 (37) | 41 (42) | 21 (21) | 0.51028 | 1.44 (0.55-3.88) | 0.12434 | 1.88 (0.80-4.62) |
| **Males** | 15 (34) | 23 (52) | 6 (14) | 17 (35) | 19 (40) | 12 (25) | 0.38834 | 1.74 (0.46-7.17) | 0.19719 | 2.09 (0.64-7.56) |
| **Females** | 13 (30) | 26 (59) | 5 (11) | 20 (39) | 22 (43) | 9 (18) | 1.00000 | 1.17 (0.27-5.48) | 0.56304 | 1.66 (0.45-6.89) |

*p*<0.05 was regarded as statistically significant, odds ratio (OR) and 95% confidence interval (CI), **^a^** AA = major allele,

**^b^** Ag = heterozygous allele, **^c^** gg = minor allele, the subjects were divided into subgroups with body mass index,

(BMI)≤25 kg/m² = **^e^** normal weight and BMI>25 kg/m² = **^f^** overweight.

(Renner et al.)
