# Supplementary material for: A Variant of the SLC10A2 Gene Encoding the Apical Sodium-Dependent Bile Acid Transporter Is a Risk Factor for Gallstone Disease
Source: PLoS One. 2009 Oct 13;4(10):e7321. doi: 10.1371/journal.pone.0007321 (PMC2757911; doi:10.1371/journal.pone.0007321)
Supplement: Table S3 — Clinical parameters in pooled Stuttgart and Aachen controls and gallstone carriers with distinct rs9514089 genotypes. Values are given as means±SEM; BMI = body mass index; a = normal range; b = in the recessive model, corrected by study centre. (0.04 MB DOC) [file pone.0007321.s003.doc]

**Supplemental: Table** **S3**. Clinical parameters in pooled Stuttgart and Aachen controls and gallstone carriers with distinct *rs9514089* genotypes

| **Subgroup** |  | **Controls** |  |  |  | **Gallstone** | **carriers** |  |
| --- | --- | --- | --- | --- | --- | --- | --- | --- |
| **Genotype** | **A/A (n=93)** | **A/G (n=129)** | **G/G (n=28)** | ***p* ^b^** | **A/A (n=91)** | **A/G (n=98)** | **G/G (n=47)** | ***p* ^b^** |
| **Variables** |  |  |  |  |  |  |  |  |
| **Age** | 60.29 (±1.36) | 63.52 (±1.12) | 62.29 (±2.33) | 0.96 | 63.34 (±1.42) | 63.52 (±1.23) | 63.06 (±1.89) | 0.84 |
| **BMI** | 25.57 (±0.45) | 25.19 (±0.33) | 25.70 (±0.63) | 0.66 | 26.49 (±0.49) | 26.10 (±0.45) | 25.96 (±0.46) | 0.66 |
| **Triglyceride (mg/dl) (1-200) ^a^** | 133.1 (±6.80) | 151.0 (±10.88) | 130.6 (±12.77) | 0.68 | 148.9 (±9.90) | 135.8 (±8.15) | 133.8 (±8.53) | 0.69 |
| **Cholesterol (mg/dl) (140-240) ^a^** | 203.1 (±4.57) | 202.4 (±4.71) | 191.4 (±9.76) | 0.24 | 199.6 (±4.92) | 206.3 (±5.08) | 187.7 (±7.34) | **0.05** |

Values are given as means ± SEM; BMI=body mass index; **^a^** =normal range; **^b^** =in the recessive model, corrected by study centre.

(Renner et al.)
